# Investigation of Physical-Mechanical Properties and Microstructure of Mortars with Perlite and Thermal-Treated Materials

**DOI:** 10.3390/ma17143412

**Published:** 2024-07-10

**Authors:** Nastasia Saca, Lidia Radu, Stefania Stoleriu, Daniela Dobre, Răzvan Calotă, Roxana Truşcă

**Affiliations:** 1 Faculty of Roads, Railways and Bridges, Technical University of Civil Engineering, 020396 Bucharest, Romania; lidia.radu@utcb.ro; 2 Faculty of Chemical Engineering and Biotechnologies, National University of Science and Technology Politehnica of Bucharest, 011061 Bucharest, Romania; stefania.stoleriu@upb.ro; 3 Faculty of Civil, Industrial and Agricultural Buildings, Technical University of Civil Engineering, 020396 Bucharest, Romania; daniela.dobre@utcb.ro; 4 Building Services Faculty, Technical University of Civil Engineering, 020396 Bucharest, Romania; razvan.calota@utcb.ro; 5 Faculty of Engineering in Foreign Languages, National University of Science and Technology Politehnica of Bucharest, 060042 Bucharest, Romania; truscaroxana@yahoo.com

**Keywords:** cement, perlite mortar, concrete demolition waste, old cement, thermally treated materials

## Abstract

This study aimed to obtain and characterize a mortar with perlite aggregate and thermal-treated materials that could substitute for Portland cement. First, the thermally treated materials were obtained by calcinating old Portland cement (OC-tt) and concrete demolition waste (CC-tt) at 550 °C, for 3 h. Second, plastic mortars with a perlite: cement volume ratio of 3:1 were prepared and tested for water absorption, mechanical strength, and thermal conductivity. The microstructure was also analyzed. Portland cement (R) was partially substituted with 10%, 30%, and 50% OC-tt. Thermal-treated materials negatively influenced the compressive and flexural strengths at 7 and 28 days. With an increase in the substitution percentage to 50%, the decrease in the compressive strength was 40% for OC-tt and 62.5% for CC-tt. The presence of 10% OC-tt/CC-tt positively influenced the water absorption. The thermal conductivity of the tested mortars was in the range of 0.37–0.48 W/m·K. SEM analysis shows the expanded perlite pores remained unbroken.

## 1. Introduction

Expanded perlite is an aggregate that offers significant benefits in terms of heat insulation of buildings. Perlite is obtained by expanding the initial volume of a natural vitreous rock called perlite rock by 5 to 20 times through intense heating (900–1200 °C) [1]. The perlite is a mesoporous material with a pore diameter in the range of 20–50 Å and an average radius of 16.535 Å [2]. The properties of concretes and mortars with perlite are described in the literature [3]. Gandage et al. [4] investigated self-compacted concrete’s fresh and hardened properties with sand substituted by perlite. The results showed that the optimum perlite content is 5% from the compressive strength perspective. A reduction in the thermal conductivity of 20.78% was obtained for concrete with 20% fly ash and 5% perlite compared to reference concrete. The study of Sengul et al. [5] on lightweight concrete with expanded perlite as natural sand (substitution percentage between 20% and 100%) revealed an improvement in thermal properties and higher water absorption with increasing perlite content. Also, a decrease in compressive strength was observed. Tie et al. [6] studied the properties of fresh and hardened mortars with expanded perlite and expanded vermiculite and observed that expanded perlite improved the flow of fresh mortar and decreased mechanical strengths. When expanded perlite replaced sand (from 0% to 15%) in cement mortar, a reduction in mechanical strength, an increase in water absorption, and an improvement in thermal conductivity were obtained [7]. Similar results were obtained by Landon and Garcia-Ruiz [8].

To obtain materials with high thermal performance, Balbuena et al. [9] prepared and tested cement-based mortars with aerogel, perlite, and vermiculite. The sample with 10% perlite and 10% aerogel outperformed all other samples in terms of thermal inertia and final insulation capacity. Materials based on perlite were also studied for controlling indoor temperatures and humidity, and the results showed that expanded perlite cement mortars work well for indoor wall applications [3].

Currently, there are many studies on replacing cement with alternative binders and supplementary cementitious materials. Research on the use of construction and demolition wastes as substitutes for cement and aggregate is promising for EU member states due to the limited availability of traditional supplementary cementitious materials including fly ash, granulated blast furnace slag, and natural pozzolans, and concerns regarding the conservation of natural resources [10,11]. Crushed concrete waste and perlite powder were used by Dacic and Fenyvesi [12] to investigate the evolution of the packing density of cement paste. The results revealed that waste perlite and recycled concrete powders increased the packing density at the water/cementitious material ratio of 0.19. Since the researchers observed that concrete regains mechanical strength when cooled with water, after heating, many studies on the composition and characteristics of dehydrated cement pastes and concretes prepared in the laboratory were developed [13,14,15]. In the thermal treatment of Portland cement paste or fine concrete waste, two essential temperatures were identified [16]: 450° (portlandite dehydrates to form quicklime) and around 650 °C (calcite decomposes to produce CO_2_). As a result, the recycled cement may comprise anhydrous cement, remaining cement hydration products, and partially dehydrated components, depending on the temperature [17]. The kind of precursor and treatment temperature determine the composition of recycled cement. According to Bogas [14], free lime (CaO), calcite (CaCO_3_), and larnite (C_2_S) are the main components. The rehydration capacity of recycled concrete depends mainly on the source and fineness of the material [18], the thermal treatment temperature, and the substitution percentage of the cement [19,20].

Also, numerous studies have shown that particle size impacts recycled concrete’s chemical and physical characteristics. According to Florea et.al. [21], 70% of particles contained hydrated and anhydrous clinker when cement granularity was below 80 µm. A higher amount of silicon oxide was presented when the particles were larger than 80 µm. Kwon et al. [22] evaluated clinkering by free CaO, XRD and thermal analysis, compressive strength, and flowability and found overall properties to be at least 80% of OPC or higher. He et.al. analyzed CO_2_ emissions from OPC and recycled cement manufacturing [23]. The study found that recycled cement processed at 450 °C reduced CO_2_ emissions by over 94% compared to standard Portland cement, and significantly less than recycled cement heated at 800 °C [23]. The compressive strength of recycled cement-based materials significantly decreased in comparison with the original cement paste, regardless of testing time [17]. 

The production of recycled cement was analyzed from two points of view: the possibility of reducing CO_2_ emissions into the atmosphere as a result of the powder’s decarbonation, and the energy consumption for obtaining 1 kg of the binder. Analysis by Gastaldi et al. [24] of recycled cement obtained from cement paste showed a diminishing of CO_2_ released when ordinary Portland clinker was substituted by the burnt mix with 55% wt. cement waste. A significant reduction in CO_2_ emissions was obtained by Tokareva et al. [25]. Their study also shows that the thermal treatment of wastes at 400 °C and 500 °C increased the energy consumption and significantly improved the mechanical strengths, without recording CO_2_ emissions and energy consumption considerably greater than that for samples with untreated wastes. 

An analysis by Sousa and Bogas [26] assessed a reduction in CO_2_ emissions between 22% and 29% when magnetic separation is used in the recycled cement production process to separate the cement from the aggregates. Also, it has been shown that the amount of energy used is less than that needed to produce Portland cement. However, the requirement of washing and drying the material prior to magnetic separation indicates a 60% increase in thermal energy usage over the average for the production of Portland cement.

The idea of this study was to create an expanded perlite mortar with recycled materials that would enhance the energy efficiency of buildings and save natural resources. Preparing and characterizing thermally treated materials was the first step in this direction. In the second step, the behavior of the mortars in their fresh state was evaluated; in the third step, the hardened mortars were examined using tests for density, water absorption, compressive strength, flexural strength, and thermal characteristics using λ-value analysis. Additionally, the microstructure of mortars was examined. 

## 2. Materials and Methods

**Figure 1:**
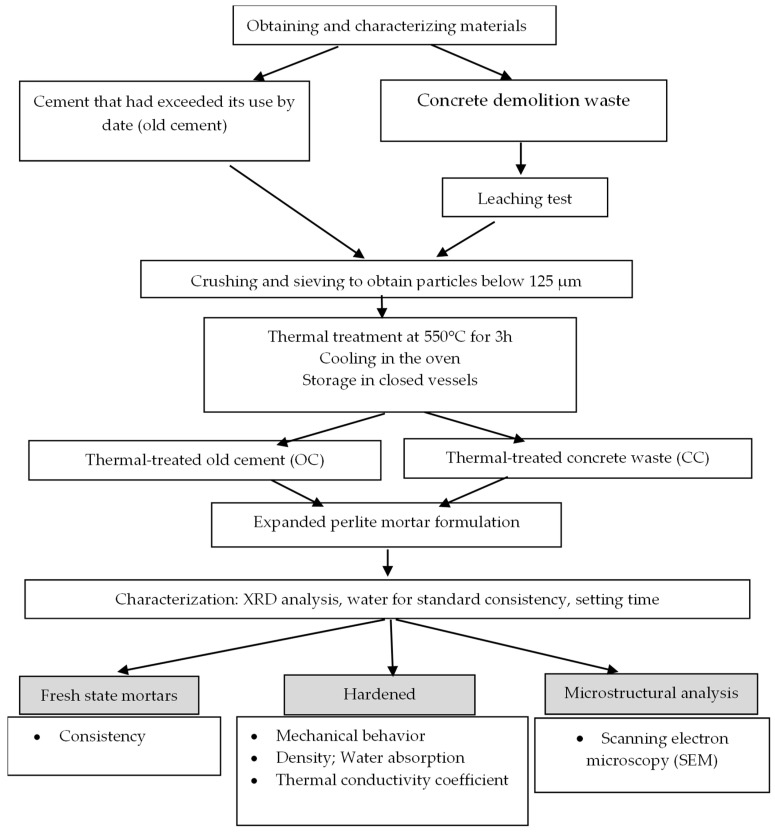
Work methodology.

The chosen approach for this study is described in Figure 1.

### 2.1. Materials

**Figure 2:**
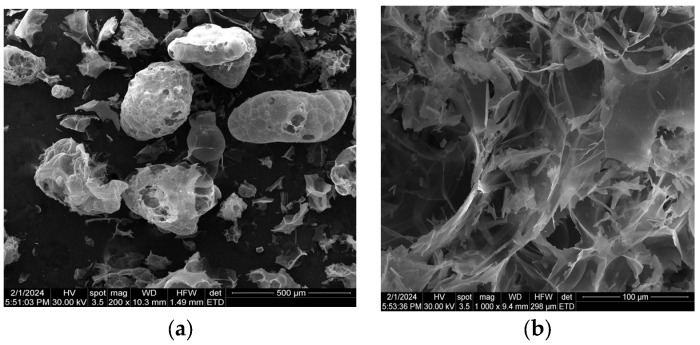
SEM images of perlite beads (**a**) and of the perlite structure (**b**).

Expanded perlite with sizes smaller than 2 mm, of the type IZO-PER 2 (producer S.C. Procema Perlit SRL, Jilava, Romania), was used in this study (Table 1). SEM images of this perlite reveal a foam-like cellular structure (Figure 2).

Portland cement type CEM II/A-LL 42.5 R, produced by Heidelberg Materials (Heidelberg, Germany), was used as the reference. The cement was partially substituted by calcinated old cement and calcined concrete demolition waste.

**Figure 3:**
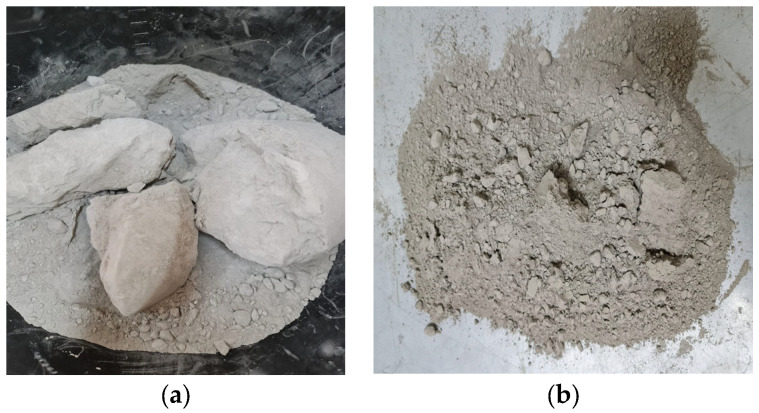

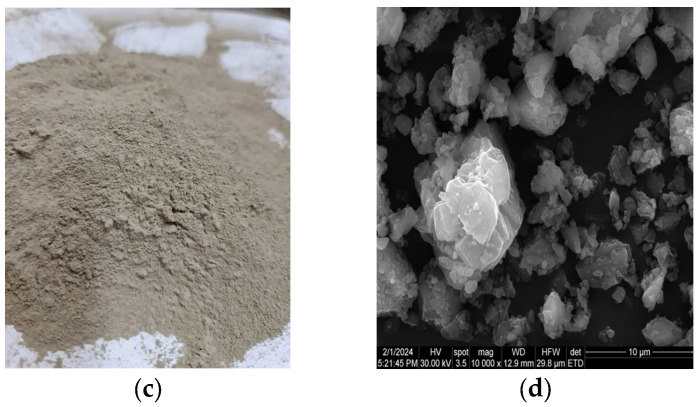
Images of old cement: initial (**a**), ground and sieved old cement (**b**), after thermal treatment (**c**), and SEM image of thermally treated old cement (**d**).

The old cement was CEM II/A-LL 42.5 R kept for 2 years in laboratory conditions. During that time, the cement partially hydrated and appeared as agglomerations of different sizes. A planetary ball mill reduced the old cement to particles under 125 µm. After this, the old cement went through thermal treatment at 550 °C, with a residence period of 3 h at the maximum temperature. The material was cooled inside the oven until it reached ambient temperature. The images of the old cement and the thermal-treated cement are presented in Figure 3.

The concrete demolition waste was crushed with a jaw crusher and sieved until the material was smaller than 4 mm. After that was tested to assess compliance with the criteria mentioned in Decree no. 95/2005 for inert wastes [27], as can be seen in Table 2. A leaching test was performed according to SR EN 12457:2003 [28,29]. The electrical conductivity of the eluates was 626 µS/cm for L/S = 2, and 252 µS/cm for L/S = 10.

**Figure 4:**
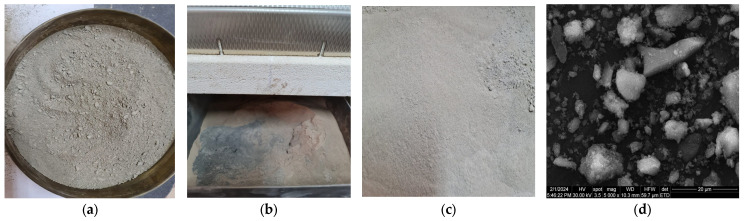
Images of concrete demolition waste: waste under 4 mm (**a**), after thermal treatment (**b**,**c**), SEM image of calcined concrete (**d**).

This material was ground with a planetary ball mill and sieved with a 125 µm sieve. The obtained powder was thermally treated and cooled in the same conditions as the old cement. Images of the concrete demolition waste at different stages of preparation are shown in Figure 4. The particles had generally angular shapes.

Materials were thermally treated in a Nabertherm N11/H electric oven (Nabertherm, Lilienthal, Germany). The heating rate was approximately 20 °C/min. After cooling, the materials were kept in closed containers until use.

The following processes are usually considered as taking place during the heat treatment of hydrated cement [13,20,30,31]:

- Evaporable water and some bound water evaporate between 20 and 130 °C.
- Ettringite and gypsum dehydrate between 110 and 200 °C.
- Calcium silicate hydrates and carboaluminate hydrates dehydrate between 140 and 450 °C.
- Portlandite dehydroxilation and α-C_2_S are formed by calcium silicate depolymerization between 450 and 650 °C.

From the compositional point of view, the reference cement, the old cement, and the untreated concrete waste/thermal-treated waste were characterized by x-ray diffraction analysis. 

**Figure 5:**
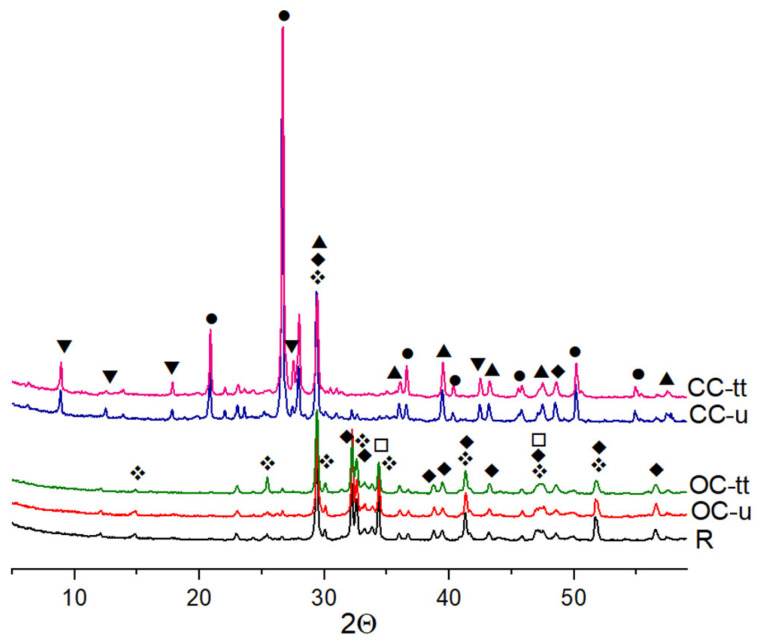
XRD patterns for reference (R), untreated old cement (OC-u), and concrete waste (CC-u) and thermally treated materials (OC-tt and CC-tt). Codes: ▼—ettringite, ●—quartz, ▲—calcite □—portlandite, ❖—C_3_S-tricalcium silicate, ♦—C_2_S-dicalcium silicate.

The XRD patterns presented comparatively in Figure 5 show that the main components of concrete waste are quartz and calcite. These compounds were derived from aggregates and did not transform during thermal treatment. The presence of the calcite could be due to the concrete carbonization process over time.

The untreated old cement contained C_3_S, C_2_S, calcite, ettringite, and portlandite. Calcium oxide was not identified as a cement/concrete waste calcination product. 

During thermal treatment, decomposition of ettringite, portlandite, and C-S-H occurred. As in the case of portlandite, dehydrated C-S-H hydrates if the curing medium has sufficient moisture [17]. The selected temperature was not high enough to produce calcite decarbonization. Calcite particles can react with cement components and form carboaluminate hydrate, and also have both filler and nucleation effects. 

The main characteristics of thermal-treated materials are presented in Table 3.

The higher water for standard consistency of OC-tt, compared to R, can be explained by a possible higher fineness. Carrico et al. found that thermoactivated cement particles agglomerate, reducing their fineness [33]. Regarding setting time, a stiffening of the CC-tt paste was observed after 24 h, which was later found to correlate with the XRD analysis results. Results from previous studies have shown that recycled cement reactivated by thermal treatment generally has a shorter setting time compared to ordinary Portland cement. Xu et.al. [30] suggest that this could be related to a false setting phenomenon caused by the quick reaction of free lime and water, resulting in the synthesis of portlandite in the absence of gypsum. 

In thermally treated OC, no patterns were observed for C_2_S polymorphs, a result of C-S-H dehydration with a broad peak between 30° and 35° (2theta). Its absence was expected because this phase typically formed at higher heating temperatures, according to research [18,19,20,33,34].

**Figure 6:**
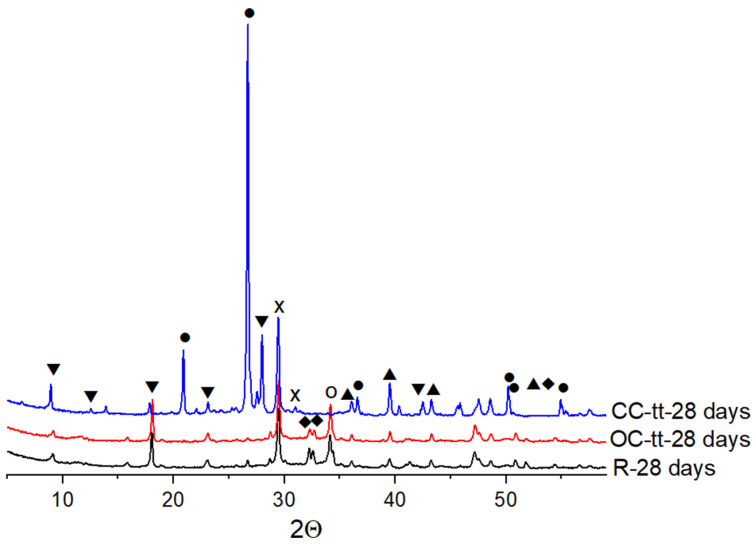
XRD patterns of 28 days hardened pastes of reference (R), calcinated old cement (OC-tt), and calcinated concrete waste (CC-tt). Codes: ▼—ettringite, ●—quartz, ▲—calcite, X—CSH, O—calcium hydroxide.

The XRD patterns of pastes with the reference cement, CC-tt, and OC-tt were measured at a hydration time of 28 days (Figure 6). The same phases were detected in the reference cement and OC-tt hardened pastes: ettringite, calcium hydroxide, C-S-H, and calcite. The hardened paste of CC-tt mainly consisted of quartz and calcite. 

### 2.2. Mixing, Molding, and Curing Conditions

The mortars contained CEM II/A-LL 42.5R partially substituted (0%, 10%, 30%, and 50%) by thermally treated OC and CC. Expanded perlite as aggregate was used in a volume ratio of cement: perlite of 1:3. The mortars were prepared with drinking water, with the water/cement ratio varying from 0.53 to 0.61, to obtain a mortar with consistency between 140 and 200 mm (Table 4).

The mortars were mixed mechanically in a Tecnotest mixer. After mixing, the mortars were poured into molds of size 160 × 40 × 40 mm and molds of size 300 × 300 × 60 mm. The samples were compacted by vibration. The molds were covered with a polyethylene film and kept in laboratory conditions for 24 h. After that, the samples were demolded and cured at 20 °C and a relative humidity of 95 ± 5% until testing time. 

### 2.3. Methods

#### 2.3.1. Mortars in Fresh State

The consistency of mortars was tested by the flow table method [35]. A truncated mold was filled with mortar in two layers, each of them tamped down 10 times to ensure uniform filling. The excess mortar was removed and the mold was lifted away. Immediately, the table was dropped 15 times. The average diameter was measured with a caliper. 

#### 2.3.2. Mortars in Hardened State

##### Water Absorption

Three mortar samples with the age of 28 days were dried in an oven at 105 °C until a constant weight, m_i_, was obtained. The specimens were kept in a desiccator until they reached the ambient temperature and then sealed to limit the passage of water to one face using a hydrophobic coating, according to SR EN ISO 151548:2004 [36]. The samples were partially immersed in water at a depth of 5 ± 2 mm, and the water level was kept constant. The mortars were weighed at 20 min, 1 h, 2 h, 4 h, 8 h, and 24 h after immersing, and the weights recorded in m_t_. The gain in mass was reported for the surface of samples in contact with water, *A*:

(1) $$\Delta m_{t}=\frac{m_{t}-m_{i}}{A}\;(kg/m^{3})$$

##### Mechanical Strengths

Mortars were evaluated for compressive strength at the ages of 7 and 28 days. To find the average compressive and flexural strength, three specimens 40 × 40 × 160 mm were tested at each age [37]. Before loading, the samples were positioned perpendicular to the casting direction. Hydraulic testing machines with load capacities of 300 kN and 3000 kN were used to test all samples. 

##### Thermal Conductivity

The thermal conductivity was determined using a heat transfer service unit for building and insulating materials, type H111N. The mortar boards were placed between a hot plate and a heat flowmeter attached to a cold plate. The entire unit was well insulated, and the measurements were recorded when the average temperature corresponding to each plate remained constant or, in other words, when a steady-state regime was attained. The tests were performed according to EN 12667:2001 [38].

##### X-ray Diffraction

X-ray diffraction was performed with a SHIMADZU XRD 6000 diffractometer (Shimadzu, Kyoto, Japan) with Ni-filtered Cu Kα radiation (λ = 1.5406 Å), with a 2θ range of 5–60°, a scanning speed of 2°/min, and a step size of 0.02 min/step. 

##### Microstructure of Mortars

SEM analysis using an FEI Inspect F scanning electron microscope (FEI, Hillsboro, OR, USA) equipped with an energy dispersive x-ray spectrometer was used to obtain information regarding the microstructure of 28-day mortars. Before imaging, a thin conductive coating (gold) was added to the samples. 

## 3. Results and Discussion

### 3.1. Mortars in Fresh State

**Figure 7:**
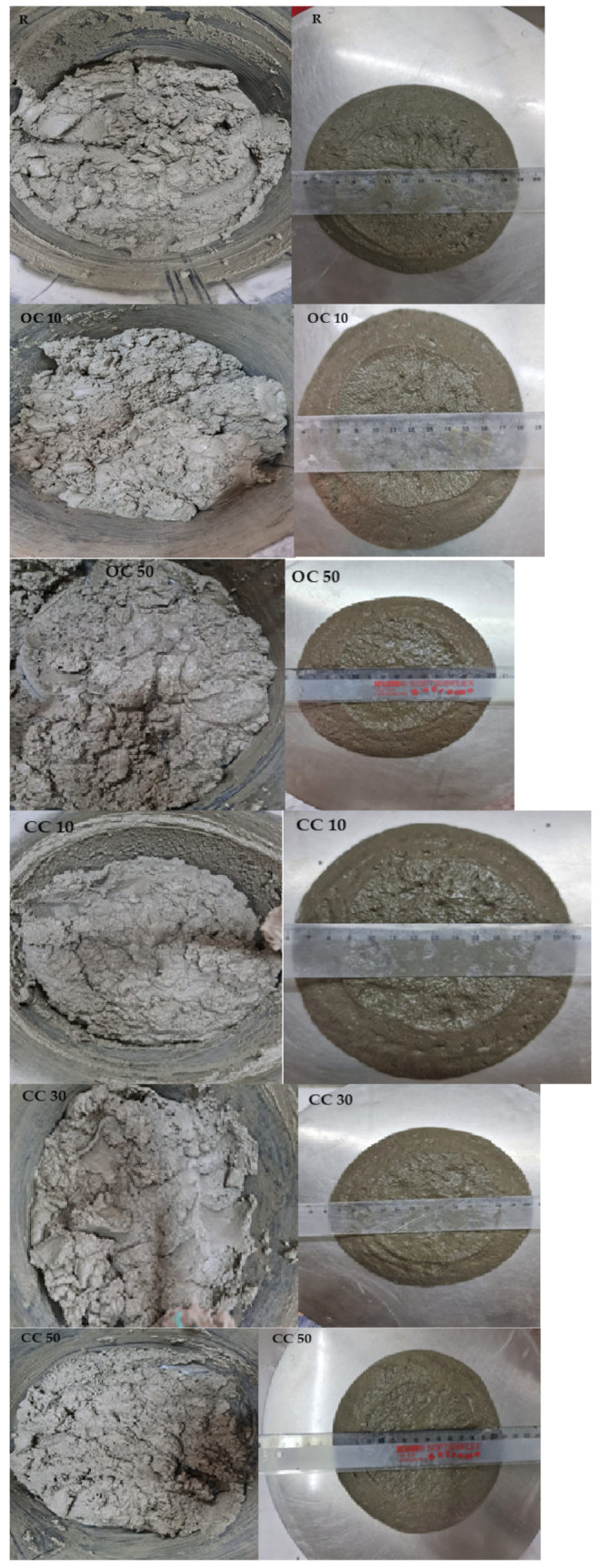
The images of some fresh mortars after mixing and during the flow table test.

The consistency of mortars was assessed with the flow table. The assumed flow was within the 141–159.5 mm range, which indicates mortars with plastic consistency (140–200 mm [35]). The water content slightly increased with the increase in the percentage of substituted cement. The visual examination of the aspect of the fresh mortar suggested stable mortars. The images of some mortars immediately after mixing and during the measurements of flow diameter are given in Figure 7.

### 3.2. Water Absorption

The density of hardened mortars dried in the oven was between 1332 and 1027 kg/m^3^; the lowest values were obtained for mortars with 50% OC-tt and 50% CC-tt.

At the beginning of the test, water absorption increased quite rapidly. The water absorption speed appeared to be similar for all samples, and the variation in water absorption per unit area was linear (R^2^ was higher than 0.94 for all samples, and the highest values were obtained for mortars with 50% OC-tt and 50% CC-tt (0.9879 and 0.9885, respectively). 

Due to the perlite-specific porous structure, the pore size distribution influences the capability of mortar to absorb water. In the initial stage of the process, the water is rapidly pulled into the pores. The smaller capillary pores will be filled with water, whereas the weaker capillary suction in the larger pores slows water absorption. According to Silva et.al. [39], the pores of expanded perlite work as air voids and limit the capillary effect, resulting in a significantly greater influence of this aggregate on the capacity to absorb water than on absorptivity. A study by Xiong et al. [40] showed that expanded perlite thermal insulation mortar has three pore sizes: 0.01–0.1 μm, 0.1–10 μm, and 10–1000 μm. The pores smaller than 10 μm have an impact on capillary water absorption, while pores smaller than 0.1 μm govern vapor adsorption. The pores with the ability to absorb free water have a range diameter between 0.01–10 μm [41].

**Figure 8:**
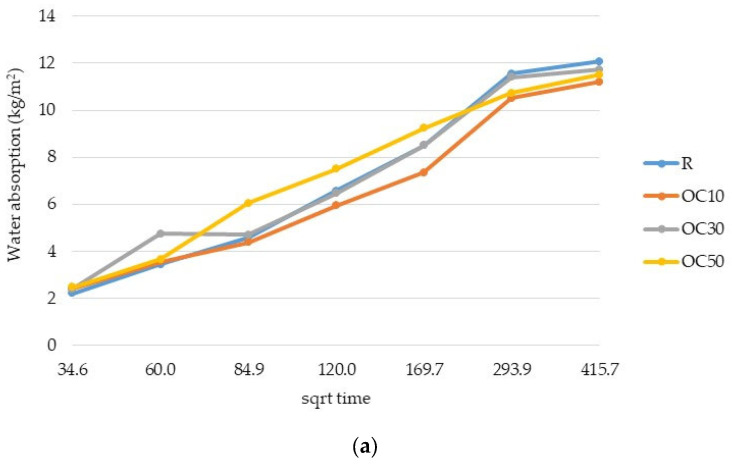

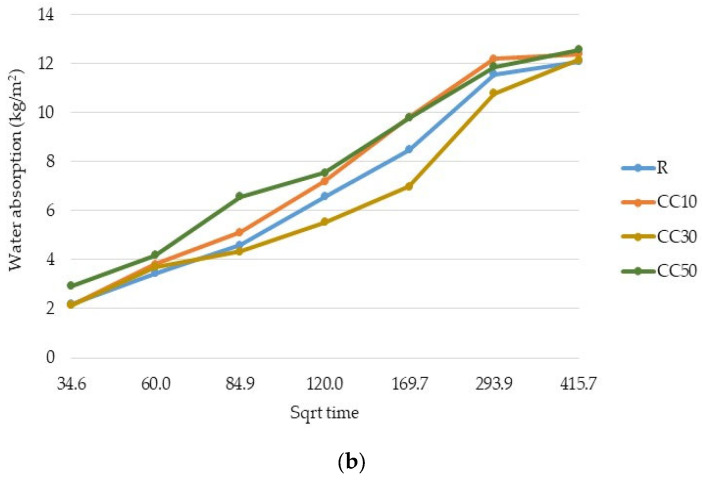
The water absorption of mortars with OC-tt (**a**) and CC-tt (**b**).

The replacement of reference cement by OC-tt positively influenced the water absorption after 2 h (Figure 8a). After 24 h, the mortar with 10% OC-tt had lower water absorption, which suggests a more compacted structure. 

In the case of mortars with CC-tt, CC10 had higher water absorption than the reference (12.2 kg/m^2^ compared to 11.6 kg/m^2^, after 24 h). A smaller value (10.8 kg/m^2^) was recorded for CC30 (Figure 8b).

After quick absorption, the water enters samples through the capillary pores. After that, water keeps moving into the gel pores of the material, based on the diffusion mechanism [42,43].

After 48 h, the mortars with 30% and 50% OC-tt had slightly higher water absorption than OC 10 and were comparable to the reference. In the case of mortars with CC-tt, the water absorption was comparable to the reference mortars, regardless of the substitution percentage of the cement.

### 3.3. Mechanical Strengths

**Figure 9:**
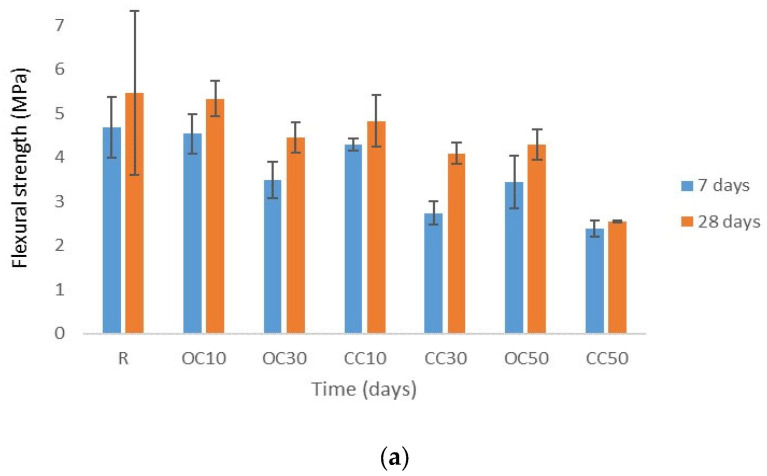

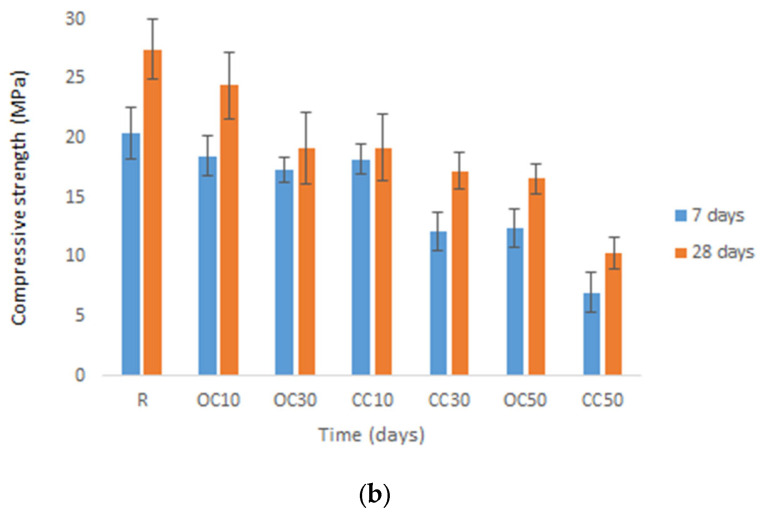
Flexural strength (**a**) and compressive strength (**b**) of mortars.

Figure 9 shows the flexural and compressive strength test results. At 7 days, the reference mortar had the highest flexural strength. Over 28 days, the mortar with 10% OC-tt developed a flexural strength of 5.33 MPa in comparison to 5.47 MPa for R. Replacing cement with thermally treated materials negatively affected the 28-day mechanical strengths. 

The mortars with CC-tt had lower mechanical strength than the reference, the difference being significant for 50% CC-tt. These results are supported by the absence of key phases in the concrete waste for the thermal activation process, namely portlandite and ettringite. Heating the concrete waste at 550 °C did not modify its chemical composition.

The mechanical strengths are more influenced by the type of thermally treated materials that substitute for cement than the quantity of water used to obtain mortars with similar consistency, as can be seen by comparing the mechanical strengths of mortars OC10 and CC10 (similar water/cement ratio).

When reference cement was replaced with 10% OC-tt, the compressive strength reached 18.5 MPa at 7 days and 24.4 MPa at 28 days, which is lower than the compressive strength obtained for the reference mortar. The ratio between the compressive strengths of mortars with OC-tt and the reference mortar became significantly higher when the substitution percentage of the cement increased up to 50%, from 88.9% for OC10 to 70% for OC30 and 60% for OC50. The results show that calcined old cement was less effective when the substitute cement content was more than 10%. The 7-day flexural strengths varied between 3.44 MPa and 4.53 MPa for mortars with OC-tt and increased at 28 days, with 4.30 and 5.33 MPa values. The flexural strengths decreased with an increase in OC-tt content.

The same trend was also observed for mortars with cement partially substituted by CC-tt. For 28 days, the compressive strength was 19.2 MPa for CC10, 17.2 MPa for CC30, and 10.3 MPa for CC50. In comparison with the reference mortar, the ratio of the 28-day compressive strength of mortars with CC-tt to the compressive strength of the reference mortar was in the range of 70% and 38%. The 28-day flexural strengths varied from 2.55 MPa to 4.83 MPa, the lowest value being recorded for mortar with 50% CC-tt. The diminishing mechanical strengths were significant for mortars in which cement was substituted by CC-tt. The weak performance of the tested mortars was directly associated with the proportion of cement.

To assess the reliability of the results related to mechanical strength, the standard deviations were also calculated, the graphic representations being customized according to them. Because some standard deviation error bars overlap, meaning that the difference between means could not be significant, especially for the flexural strength, the statistical test-t was applied to check. Since the alpha value was considered 0.05 and the *p*-value was less than 0.05, there was a statistically significant difference between the means of the data obtained at 7 days and at 28 days.

**Figure 10:**
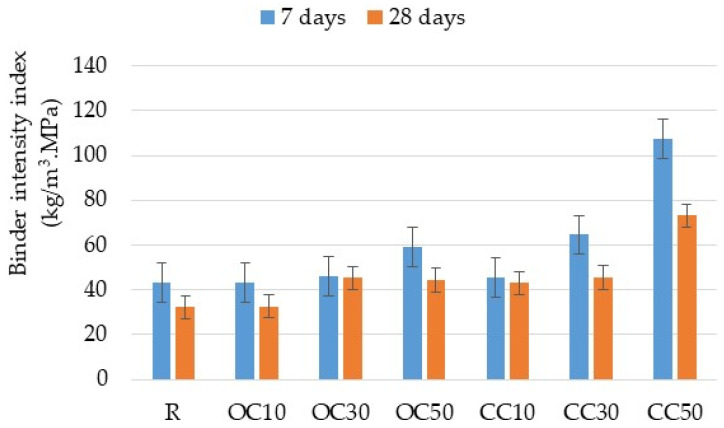
The binder intensity index for mortars prepared with cement and thermal-treated materials.

Based on the cement content of each mortar (kg/m^3^) and the compressive strength, a binder intensity index was calculated (Figure 10). A binder intensity index describes the effectiveness of utilizing binder materials by quantifying the amount of binder required to obtain 1 MPa of compressive strength.

The analysis of the binder intensity revealed a progressive decrease in their value with time. An increase in the substitution percentage of the reference cement led to a rise in the binder intensity index, the trend being significantly higher for 30% and 50% CC-tt. 

### 3.4. Thermal Conductivity of Mortars

The thermal properties are directly related to the density, composition, and air gaps in the mortars. The thermal conductivity of mortars, expressed by the λ-value in W/m·K, is significantly higher than that of the air. Expanded perlite aggregate has a lot of air cells, which provide it with great insulating properties. It is noteworthy that compared to conventional sand plaster, expanded perlite plaster provides up to 4–6 times greater resistance to heat transmission [44].

The thermal conductivity of the tested mortars was in the range of 0.37–0.48 W/m·K. The highest value was obtained for the reference mortar, which suggests some influence of the cement type (0.48 W/m·K). Mortars with 10% and 30% OC-tt had 0.46 W/m·K and 0.37 W/m·K, respectively. Mortars with 10% and 30% CC-tt had λ-values of 0.42 and 0.45 W/m·K, respectively. The values suggest that the mortars with thermal-treated materials had higher porosity than the reference. It is well established that as porosity increases, the thermal conductivity decreases. The air in the pores is a great insulator, enhancing the composite’s thermal insulation properties.

### 3.5. The Microstructure of Mortars

**Figure 11:**
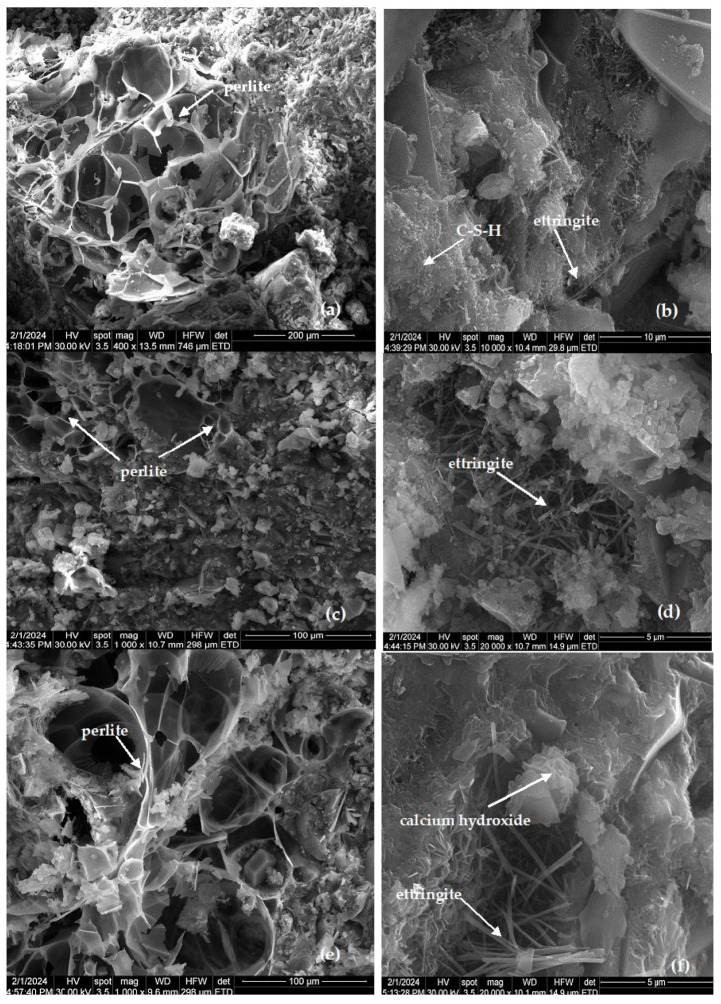
Microstructure of perlite mortars: reference (**a**,**b**); mortar with 30% OC-tt (**c**,**d**) and 30% CC-tt (**e**,**f**).

The scanning electron microscopy (SEM) images of samples hardened for 28 days provide compelling evidence of the tight integration of expanded perlite within the cement matrix, as depicted in Figure 11. Notably, there is no observable splitting within the examined samples. Moreover, the SEM images illustrate that the pores of the expanded perlite remain intact (Figure 11a), indicating that the addition of components and the mixing process were conducive to preserving the structural integrity of the perlite.

A closer examination through SEM analysis unveils crystallized structures of the hydration compounds within the samples (Figure 11b–f). These results are in good agreement with the results of the XRD analyses (Figure 6). Across mortar compositions, the presence of elongated and rounded needle-like crystals of ettringite is evident, suggesting a consistent formation of this mineral. Additionally, calcium silicate hydrates are observed in the form of crumpled foils, indicative of their characteristic morphology within the cementitious matrix. Furthermore, hexagonal crystals of Ca(OH)_2_ are discernible, contributing to the overall mineral composition of the specimens. This comprehensive analysis underscores the intricate interplay between the constituent materials and highlights the structural stability achieved within the mortars.

## 4. Conclusions

The potential use of calcinated old cement and concrete demolition waste as recycled cement has been investigated in this study. The XRD diffraction analysis of anhydrous materials and pastes made from R, OC-tt, and CC-tt hardened for 28 days highlighted that thermal-treated concrete demolition waste contained mainly quartz and calcite. Expanded perlite mortars with cement partially substituted by thermally treated materials (0%, 10%, 30%, and 50%) were prepared to investigate this application. 

Overall, the following considerations can be made:

- The water absorption and mechanical strengths of the mortars were influenced by the type and content of calcinated materials.
- The replacement of cement type CEM II/A-LL 42.5R with thermal-treated old cement decreased the 28-day compressive strength of expanded perlite mortar from 11% for 10% OC-tt up to 40% for 50% OC-tt. After 48 h, the water absorption of mortars with 30% and 50% OC-tt was 11.72 kg/m^2^ and 11.50 kg/m^2^, respectively, in comparison with 12.3 kg/m^2^ for the reference mortar.
- The results showed that mortars with CC-tt presented lower efficiency than those with OC-tt and CEM II/A-LL 42.5R. This observation is related to the lower quantity of cement in CC-tt, emphasizing how crucial it is to separate the aggregate from cement paste in concrete demolition waste. The decrease in 28-day compressive strength was in the range of 30–62.5%, the higher diminishment corresponding to 50% CC-tt. At 48 h, the water absorption was comparable to the reference mortar (12.14–12.55 kg/m^2^).
- The thermal conductivity coefficient of the mortars with thermal-treated materials was between 0.37 and 0.48 W/m·K. The reference had the highest λ-value.
- The SEM images of the mortars revealed the presence of ettringite crystals, calcium silicate hydrates as crumped foils, and hexagonal portlandite crystals. The expanded perlite pores were unbroken.
- A study on the influence of the storage conditions of thermally treated materials on their composition is necessary to identify the maximum storage time in closed vessels, under laboratory conditions.

## Figures and Tables

**Table 1:** Properties of perlite.

Colour	Light Grey
Bulk density (SR EN 1097-3/2002)	40–65 kg/m^3^
Size distribution (SR EN 933/2002)	0–2 mm, max 10% 0.5 mm
Thermal conductivity coefficient (SR EN 12667/2002)	max 0.042 W/m K
Compaction resistance (SR EN 13055-1/2003)	Min. 0.07 N/mm^2^
Fire reaction class	A1

**Table 2:** Values of concentration as measured in eluates and reference values (Decree no. 95/2005).

	L/S Ratio			
	2		10	
	Concentration (mg/kg)	Reference Value (mg/kg)	Concentration (mg/kg)	Reference Value (mg/kg)
As	0.012	0.1	0.05	0.5
Ba	0.064	7	0.17	20
Cd	<0.002	0.03	<0.01	0.04
Cr total	0.004	0.2	0.04	0.5
Cu	0.13	0.9	0.69	2
Hg	<0.0001	0.003	<0.0005	0.01
Mo	0.036	0.3	0.05	0.5
Ni	0.008	0.2	0.03	0.4
Pb	0.006	0.2	0.02	0.5
Sb	0.01	0.02	0.02	0.06
Se	<0.002	0.06	<0.01	0.1
Zn	0.052	2	0.34	4
Fluorides	2.001	4	5.998	10
Phenol index	<0.2	0.5	<1	1

**Table 3:** Characteristics of calcined materials and cement CEM II/A-LL 42.5R.

	Old Cement Thermal Treated (OC-tt)	Concrete Waste Thermal Treated (CC-tt)	CEM II/A-LL 42.5R (R)
Bulk density (kg/m^3^)	883	991	1113
Water for standard consistency (%) SR EN 196-3 [32]	0.38	0.34	0.31
Setting time (min.)			
SR EN 196-3 [32]			
initial	190	>24 ore	135
final	295	>100 ore	195
Compressive strength (MPa) on paste, 7 days	31.4	0.16	47.2

**Table 4:** Composition, consistency, and density of dry hardened mortars.

Mortar Code	CEM II/A-LL 42.5R (kg)	OC-tt (kg)	CC-tt (kg)	Water/Cement	Consistency (mm)	Density of Dry Mortars (kg/m^3^)
R	100			0.53	142	1332
OC10	90	10		0.55	141	1317
OC30	70	30		0.56	144	1260
OC50	50	50		0.61	152	1171
CC10	90		10	0.56	147	1315
CC30	70		30	0.61	141	1254
CC50	50		50	0.69	159.5	1027

## Data Availability

Data are contained within the article.
